# Supplementary material for: Time‑resolved multi-omic analysis of paclitaxel exposure in human iPSC‑derived sensory neurons unveils mechanisms of chemotherapy‑induced peripheral neuropathy
Source: Cell Death Dis. 2026 Feb 10;17(1):211. doi: 10.1038/s41419-026-08445-2 (PMC12921266; doi:10.1038/s41419-026-08445-2)
Supplement: Supplementary file 15 — Dataset 1. [file 41419_2026_8445_MOESM15_ESM.zip › CDD_Open_Data/04_RNA_Seq/Timeline_panel_plots_etc_2023-01-23_1609.zip!/Panel_Plots_etc/Evidence_Plots/72h/evidence_plot_standard contrasts_msigdb_reactome_72h_PTX_vs_DMSO_ID0_M16227_REACTOME_CHOLESTEROL_BIOSYNTHESIS.pdf]

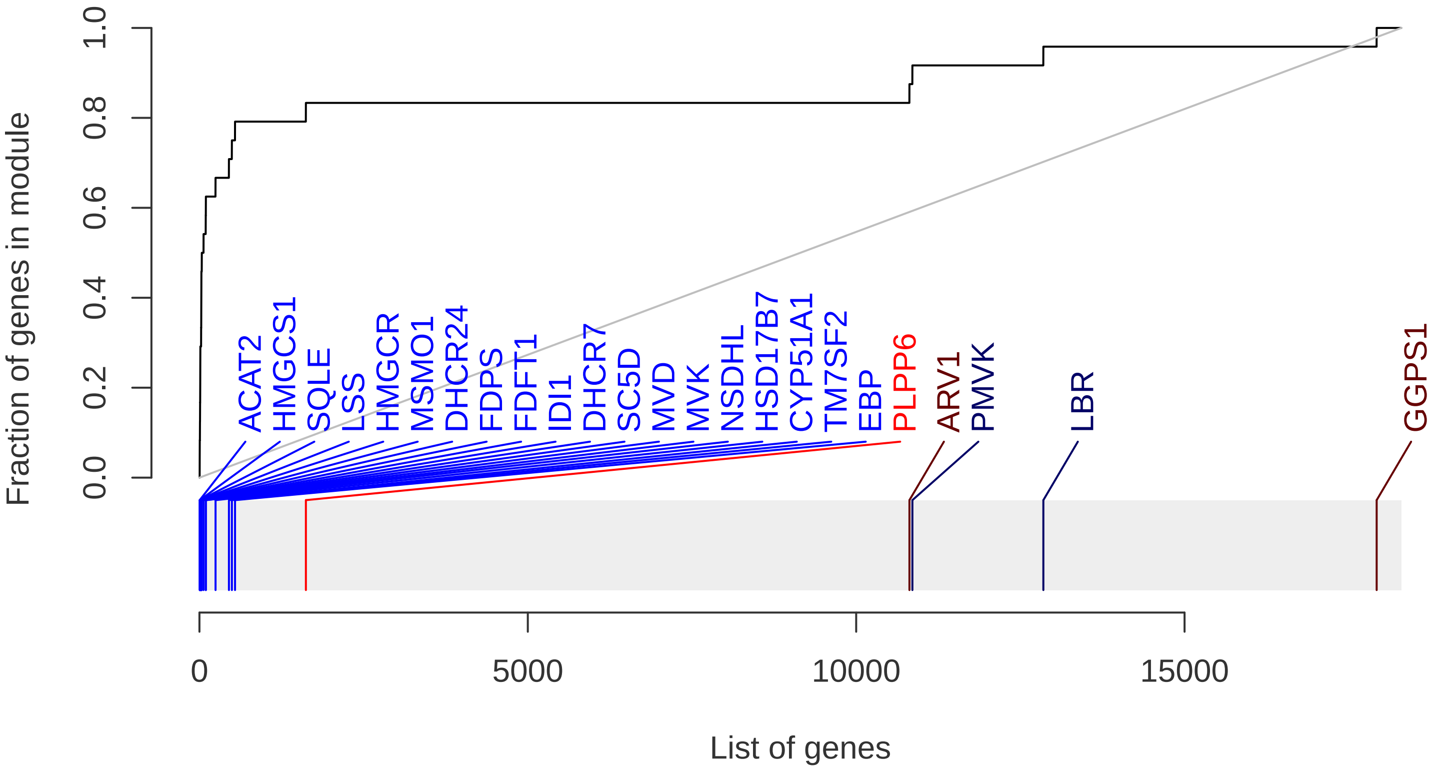

Fraction of genes in module
0.0
0.2
0.4
0.6
0.8
1.0
ACAT2
HMGCS1
SQLE
LSS
HMGCR
MSMO1
DHCR24
FDPS
FDFT1
IDI1
DHCR7
SC5D
MVD
MVK
NSDHL
HSD17B7
CYP51A1
TM7SF2
EBP
PLPP6
ARV1
PMVK
LBR
GGPS1
0
5000
10000
15000
List of genes
